# Oral Health Knowledge and Behavior among Adults in the United Arab Emirates

**DOI:** 10.1155/2019/7568679

**Published:** 2019-02-06

**Authors:** Eman Abu-Gharbieh, Basema Saddik, Mohammed El-Faramawi, Samer Hamidi, Mohammad Basheti

**Affiliations:** ^1^Clinical Sciences Department, College of Medicine, University of Sharjah, UAE; ^2^Department of Pharmacology and Toxicology, Dubai Pharmacy College, UAE; ^3^Department of Family and Community Medicine, College of Medicine, University of Sharjah, UAE; ^4^Department of Epidemiology, College of Public Health, University of Arkansas for Medical Sciences, USA; ^5^Department of Public Health, National Liver Institute, Menofiya University, Cairo, Egypt; ^6^School of Health and Environmental Studies, Hamdan Bin Mohammad Smart University, UAE; ^7^Faculty of Dentistry, Jordan University of Science and Technology, Jordan

## Abstract

**Background:**

The prevalence of periodontal diseases is increasing in the United Arab Emirates (UAE) despite a worldwide decline in the prevalence of dental caries among children and adolescents. The aims of this study were to determine the levels of oral health knowledge and health-related behavior in adult UAE residents, and the relationship between these variables and oral health.

**Methods:**

A descriptive cross-sectional survey with nonprobability sampling was used in this study. A sample of 630 adults residing in the UAE completed an oral health survey to assess their oral health knowledge and behavior. Mean oral health knowledge and behavior scores were calculated and correlated with population demographic and behavioral variables.

**Results:**

Participants were found to have an acceptable oral health knowledge score (OHKS) of 10.50 (2.36) where 62 % of participants answered the questions correctly. Results showed that age, gender, nationality, smoking, and physical activity were significantly associated with the knowledge score. However, only gender, nationality, and smoking predicted oral health knowledge scores after further regression analysis. On the other hand, the mean oral health behavior score (OHBS) for all participants was 8.91 (2.29); 98% of all participants practiced at least an acceptable level of oral behavior and 53% practiced a good to perfect level. Age, gender, educational level, employment status, insurance status, marital status, nationality, smoking, and physical activity showed significant statistical association with the score of behaviors related to oral health. Only gender, number of diabetes sessions attended, and health information sources used remained significant after further regression analysis.

**Conclusion:**

Further efforts are required to spread awareness about oral health and encourage the UAE population to develop healthy oral habits. Such programs will decrease the occurrence and burden of many chronic oral diseases especially periodontal diseases.

## 1. Introduction

The United Arab Emirates (UAE) is a federation of seven emirates and the population is made up of various demographic groups from different ethnic, cultural, and socioeconomic backgrounds. According to United Nation estimates, the population of the UAE reached 9.47 million in 2018 [1]. Demographic factors such as an aging population, high birth rate, and expatriate majority have resulted in an increase in healthcare expenditures. Health insurance coverage is universal for nationals, and laws have been instituted to ensure mandatory health insurance for nonnationals in the emirates.

The UAE health system caters for a rapid increase in population concurrent with increasing demands for health care. According to the Dubai Health Authority's (DHA) annual statistical book for 2016, Dubai has 3.4 physicians per 1000 people [2]. The proportion of dentists and nurses per 1000 people in 2016 reached 0.8 and 7.2, respectively [2]. These data indicate a gap in the number of health care professionals per population ratio especially for dental health services.

Dental health services in Dubai are provided by the governmental sector managed by Dubai Health Authority (DHA), and by the private sector. In 2016, 164,036 patients were treated in dental clinics of DHA [2], and 20% of these treatments were related to periodontitis [2].

The World Health Organization (WHO) defines oral health as “a state of being free from mouth and facial pain, oral and throat cancer, oral infection and sores, periodontal (gum) disease, tooth decay, and tooth loss [3]. Worldwide, 60–90% of school children and nearly 100% of adults have dental cavities [4].

Periodontitis is a chronic inflammatory disease in which destruction of supporting structures of the teeth occurs. Diabetes has been confirmed as a major risk factor for periodontitis, which is a possible complication of diabetes mellitus [5], and the risk of periodontitis increases by approximately threefold in poorly controlled diabetes compared to nondiabetic individuals [6]. Other risk factors that are associated with periodontitis include smoking, obesity, osteoporosis, low dietary calcium and vitamin D, stress, and inadequate coping [7, 8]. Knowledge about periodontal health and diseases, and the prevention of oral complications, including management of these conditions in patients with the abovementioned risk factors, is essential.

Lower levels of health literacy are associated with a lower understanding of the importance of prevention and maintenance, and, consequently, inferior health [9]. During the past twenty years, there has been a dramatic reduction in the prevalence of dental caries in children and adolescents, which has been mainly due to changes in living conditions, implementation of healthy lifestyles, effective use of fluoride, enhanced self-care practices, and establishment of preventive oral care programs [10–13].

Whilst several intervening factors between health literacy and oral health outcomes have been identified, knowledge remains a key component of health literacy that has received little attention [14]. The aims of this study were to determine levels of oral health knowledge and health-related behavior in adult UAE residents, and the relationship between these variables and oral health. We addressed these aims by identifying recognized risk factors that are associated with oral health, such as health-related habits, and consequences of poor oral health practices.

## 2. Methods

### 2.1. Study Design and Recruitment of Participants

A cross-sectional population survey of a nonprobability sample of adult residents of the UAE was performed. Participants over the age of 18 years who had been residing in the UAE for a minimum of one year at the time of the study (September 2015 to December 2015) were eligible. Sample size calculation with power set at 80% using t-tests for comparison of two means was used. A significance level (alpha) of 0.05 and 95% degree of confidence determined the minimum required sample size to be 310 participants. The cross-sectional design allowed us to collect observational data over a limited time period from a representative population subset.

A total of 723 participants were approached in malls and public places across the UAE. To reduce selection bias, we used cluster sampling to identify malls from different emirates within the UAE and then convenience sampling to invite participants within the malls. The malls were visited on different days at different times to ensure data collection was distributed across the days of the week and times of the day.

The aims of the study were explained to participants and verbal informed consent was obtained prior to participation.

### 2.2. Data Collection and Questionnaire Design

Data were collected through a structured interview administered questionnaire that included close-ended questions (multiple choice and true/false options). Test-retest reliability of the questionnaire was assessed by sending it on two different occasions to ten randomly selected individuals. Test-retest reliability was calculated using Spearman's correlation coefficient (r), which showed a rho-value of 0.86, implying acceptable test-retest reliability. Internal consistency for total scores showed a Cronbach's coefficient alpha of 0.73. The questionnaire was translated into Arabic and back translated, to ensure accuracy of translation, and piloted to ensure conciseness and clarity. It was also sent to a group of experts to ensure content and face validity. Questionnaires were conducted in both Arabic and English.

The questionnaire consisted of three sections with each section covering a specific domain. The first section consisted of ten questions and covered patient demographic and socioeconomic characteristics dealing with age, gender, marital status, education, employment status, income, presence or absence of dental insurance, and the type of diabetes (if present). The second section consisted of seventeen questions and captured oral health knowledge. A score of 1 reflects a correct response and a score of zero indicates an incorrect response.

An overall oral health knowledge score (OHKS) was calculated by adding each correct score for all 17 questions for each participant. Missing observations were excluded. The total scores reflect the level of knowledge and can be interpreted as follows: 0-3.99 (Very Low), 4-7.99 (Low), 8-11.99 (Acceptable), 12-14.99 (Good), and 15-16.99 (Excellent), and 17 was the maximum score which indicated a perfect level of knowledge.

The third section consisted of nine questions and captured oral health and lifestyle behaviors including frequency of dental visits, brushing, flossing, smoking, and physical activity. The behavior score was calculated by adding the scores of all six questions related to frequency of dental visits, brushing, flossing, smoking, and receipt of and compliance with oral health information for each participant. The other three questions were specific to diabetic patients. Behavior was assessed by indicating whether or not they practiced the appropriate behavior. A score between zero and one was given where one reflected the correct behavior. For certain questions related to frequencies of dental visits, brushing, flossing, and smoking scores between zero and four were used to represent the frequency of the behavior. High oral health behavioral scores (OHBS) reflected better oral health behavior. Missing observations were excluded from the analysis. The total scores reflect overall behavior and were interpreted as follows: 0-3.99 (Unacceptable), 4-8.99 (Acceptable), 9-11.99 (Good), and 12-13.99 (Excellent), and 14 was the maximum, which indicated perfect dental behavior.

### 2.3. Ethical Approval

The research protocol was approved by the Research Ethics Committee of Dubai Pharmacy College. Informed verbal consent was obtained from each participant.

### 2.4. Data Analysis

Prior to developing multiple regression models, we tested associations using t-tests or ANOVA for continuous variables to determine explanatory variables for adequate oral health knowledge and behavior. Correlations were tested using Spearman's rank order test or the biserial correlation test when the independent variable was dichotomous. We then used multiple regression analysis to model oral health knowledge and behavior. Linear regression and multiple linear regression were used. The p-value for statistical significance was set at 0.05. Data were analyzed using STATA [15].

## 3. Results

Data were collected for a total of 630 participants giving this study a response rate of 87%. Only 428 observations were analyzed for total knowledge score and 592 observations for total behavioral score as missing observations were excluded from the analysis. The majority of participants were females, 47% were between 18 and 24 years old, and almost half of the participants were Arabs. Only 12% of the participants reported having diabetes. The scores of knowledge and behavior related to oral health had a normal distribution.

### 3.1. Oral Health Knowledge

The responses to the 17 knowledge related questions were all highly correlated; therefore, they were combined into a new variable called oral health knowledge score. The 17 questions ordered by the percentage of participants who answered correctly are displayed in Table 1. The mean and standard deviation (SD) of the participants' total oral health knowledge score were 10.50 (SD=2.36); therefore, the average percentage of correct answers for the 17 questions was 62% (Table 1). Overall, participants had an acceptable level of oral health knowledge. The highest percentages of correct answers were related to questions 17 and 16, while the lowest percentages were related to questions 9 and 11 (Table 1). However, in diabetic participants, the highest percentages of correct answers were related to questions 6 and 17, while the lowest were related to questions 9 and 14 (not shown in table).

We tested for differences between cohorts within the various demographic variables. These tests showed statistically significant differences in the oral health knowledge score between smokers and nonsmokers (p=0.01), age (p=0.04), gender (p=0.03), and nationality (p=0.04) whereas no significant differences were found in oral health knowledge scores between diabetic and nondiabetic patients (p=0.75), or between physically active and inactive participants (p=0.71) (Table 2).

We then applied correlation tests to determine significant associations between the demographic and behavioral variables and the oral health knowledge score. Only gender (biserial coefficient = 0.11) and nationality (Spearman coefficient = -0.086) showed statistically significant correlation with this score with p-values of 0.035 and 0.030, respectively. Among the health behaviors, only smoking was significantly negatively correlated with the oral health knowledge score (p=0.005). The oral health knowledge score was lowest in heavy smokers and highest in nonsmokers. Bivariate regression analysis confirmed these findings.

Based on the statistically significant correlations found by correlation tests and bivariate regression analysis, we performed multiple regression analysis to determine the predictive value of the variables gender, nationality, and smoking with regard to oral health knowledge scores. The regression analysis yielded an adjusted R square of 0.90 and a p-value of <0.001, indicating that the model explained 90% of the variance in the dependent variable (oral health knowledge score).

### 3.2. Oral Health Behavior

The responses to the six behavior related questions listed in Table 3 were all highly correlated; therefore, they were combined into a new variable called oral health behavioral score. The mean behavioral score for the participants was 8.91 (SD=2.29) with maximal possible score of 14; 98% of all participants practiced at least an acceptable level of oral health behavior (score ≥ 4), and 53% practiced a good to perfect level (score ≥ 9).

About one-third of the participants reported regular dental checkups at least once a year, whereas 54% visited the dentist only when they experienced pain. Brushing teeth was practiced by 22% of participants once daily and by 79% twice daily or more frequently. Of all participants, 45% flossed their teeth once a day or more often, and 56% reported they never flossed. Seventy-six patients (12.5%) suffered from diabetes; among these, 63% had never participated in diabetic education sessions related to management of their disease, and 50% reported that they had informed their dentists that they were diabetic.

We tested for statistically significant differences in the behavioral score between categories within the various variables. The results of this analysis indicated that individuals between 35 and 44 years old had significantly higher behavioral scores than participants in other age groups (Table 4). Similarly, being female, having a graduate degree, being employed, having health insurance, being married, being local, not smoking, and being physically active were associated with a significantly higher oral health behavior score (Table 4). No significant difference between diabetic and nondiabetic participants in terms of oral health behavior (p=0.93) was found (Table 4).

We then used correlation tests to determine significant associations between demographic variables and the oral health behavior score. Gender (biserial coefficient = 0.32) and education (Spearman coefficient = 0.17) were positively correlated with the behavioral score (p-values of <0.001 and <0.001, respectively). Furthermore, physical activity, number of diabetes sessions attended, and health information sources used were also positively correlated with the oral health behavioral score with p-values of <0.001 for all. Bivariate regression analysis confirmed these results.

The variables gender, education, physical activity, number of diabetes sessions, and health information sources showed statistically significant correlations using correlation tests and bivariate regression analysis (p-values of <0.001 for all). We then performed multiple regression analysis to determine the predictive value of these variables with regard to oral health behavioral scores. The regression analysis yielded an adjusted R square of 0.92 and a p-value of <0.001, indicating that the model explained 92% of the variance in the dependent variable (oral health behavioral score).

## 4. Discussion

Results of this study have shown that participants have suboptimal oral health knowledge and behavior, consistent with other studies [16].

Participants in this study reported an acceptable level of knowledge on general concepts related to oral health, such as flossing and brushing their teeth; however, they lacked knowledge of the reasons to practice these habits, or the consequences and complications of not practicing them (gingivitis, periodontal disease, dental caries, and tooth loss). Participants also knew that they needed to keep regular twice-yearly dental appointments. They were knowledgeable about the signs of gingivitis and gum disease, and that it could lead to loss of teeth. However, participants were least knowledgeable on practices such as four-minute-long teeth brushing, the effects of poorly controlled blood glucose on developing gum disease, importance of informing their dentist if they had diabetes, and problems of teeth related to diabetes other than gum disease.

The results of this study showed that the majority of participants were aware of the relationship between diabetes and gum diseases, which is consistently higher than the reported literature [17]. This may be attributed to the increase in oral health information received in this area from health professionals due to the increase in the prevalence of diabetes both globally and in this region [18]. The increased numbers of adults diagnosed with diabetes in the UAE coupled with the recognition of gum disease as a possible complication of diabetes mellitus may explain the high levels of knowledge found amongst the Emirati participants in our study. Health professionals may have increased awareness on the importance of educating patients on the relationship between oral health and diabetes as well as emphasizing the significance of physical activity as a lifestyle factor for promoting healthy behavior.

Furthermore, our study revealed that participants were most knowledgeable on the effects of smoking on gum disease and its associated problems, such as chronic dry mouth and tooth decay. Particularly, nonsmokers had significantly better knowledge scores as well as better oral health behavior than smokers.

Demographic factors such as gender, nationality, and educational level have been documented in the literature as significant factors for differences in health literacy [19]. Our study found significant differences in oral health knowledge between the genders, with females being more knowledgeable and practicing better oral health behavior than males. Similarly, results from our study showed that individuals who did not practice healthy oral habits were more likely to have achieved lower educational levels. Our finding that the educational level was not associated with health knowledge, but that lower levels of education were associated with lower oral health behavior, is consistent with the literature [20]. Based on these findings, public health professionals need to focus on specific factors when designing oral health educational programs to increase levels of knowledge and help individuals develop healthy oral habits. These findings also suggest that dentists should assess their patients' dental knowledge of periodontal disease to tailor specific educational programs on oral hygiene, smoking, and diabetes. We observed that the youngest (18–24 years old) and oldest (older than 55 years) groups were least knowledgeable about oral health and, therefore, recommend to streamline and reevaluate oral health education programs in schools and universities and for senior citizens.

This study has several advantages; firstly, it is the first study performed in the UAE to assess oral health-related knowledge and behaviors. Secondly, we selected a representative sample from different emirates within the UAE that included local and expatriate participants, which resemble characteristics of the general UAE population.

Nevertheless, this study also has limitations. The nature of the study design was cross-sectional and prevents us from establishing causality or temporality between the study variables, knowledge, and behaviors related to oral health. Secondly, the nonprobability sampling of a convenience sample does not allow extrapolation to the whole UAE population. Only individuals who visited shopping malls or came to public spaces could be selected; this selection most likely omitted participants from rural areas. The rural population comprises 12.2% of the total population [21]. Thirdly, we had incomplete information on the important covariate income, to which only 39% of participants responded to. Income can be a potential confounder and can affect educational level and the ability to attend diabetic sessions and it can have an impact on healthy oral habits. Further research should include and encourage participants to adequately respond to this indicator. Participants in our study were not comfortable answering this question. However, despite these limitations, the results of this study can be considered important baseline data for further studies in this area.

We recommend that the health policy makers in this country encourage public health professionals to start educational programs targeted to increase awareness about oral health and encourage the population to develop healthy oral habits. Such programs will decrease the occurrence and burden of many chronic oral diseases, especially periodontal diseases. Future periodic surveys should be conducted to detect trends in oral health in the UAE population.

## 5. Conclusion

This is the first study to report information on knowledge and behaviors related to oral health in the UAE. We have designed a battery of questions to determine knowledge of oral health and practice of oral health behaviors, which we have measured in quantitative scores. We have identified regression models using a restricted number of demographic variables to predict oral health knowledge and behavior. We suggest that these simple predictive models can be used to identify individuals who can benefit from education about oral health.

## Figures and Tables

**Table 1:** Mean oral health knowledge scores for all questions answered by the participants.

Question No.	Knowledge variables for the participants	Observations	Mean	SD
17	Smoking can worsen gum disease and its associated problems, such as chronic dry mouth and tooth decay	629	0.94	0.24
16	You should keep regular twice-yearly dental appointments	630	0.91	0.29
6	Bleeding gums and red, swollen, or tender gums are signs of gingivitis and/or gum disease	624	0.87	0.33
7	Gum disease can lead to loss of teeth	625	0.82	0.39
5	Diabetes can make teeth and gum worse	624	0.77	0.42
15	Those who already have periodontitis should be concerned about their increased risk of developing serious complications from diabetes, including heart attack, stroke, and kidney disease, among others	615	0.74	0.44
2	People with diabetes are at a higher risk to have gum disease	625	0.73	0.45
1	People with diabetes are at a higher risk to have mouth infection	626	0.71	0.45
3	People with dry mouth are at a higher risk to have mouth ulcers	621	0.66	0.47
4	People with dry mouth are at a higher risk to have tooth decay	620	0.55	0.50
12	You may not be able to tell that you have serious gum disease	621	0.47	0.50
14	Gum disease is often painless	614	0.45	0.50
8	Brushing once a day for four minutes is sufficient to clean the mouth	626	0.44	0.50
10	Poorly controlled blood glucose will not affect developing gum disease	619	0.41	0.49
13	The first stage of gum disease is called periodontitis	449	0.40	0.49
11	You do not need to tell your dentist that you have diabetes	626	0.36	0.48
9	The only problem affecting gums and teeth for people with diabetes is gum disease	624	0.29	0.45
**Combined Oral Health Knowledge score**		**428**	**10.50**	**2.36**

**Table 2:** Demographic variables and their relationship with mean oral health knowledge score.

Variable	Categories	N (%)	Mean	SD	P value^*∗*^
Age	18-24	243 (56.7)	10.43	2.36	0.04
	25-34	74 (17.3)	10.80	2.47	
	35-44	51 (11.9)	10.86	2.12	
	45-54	40 (9.3)	11.73	2.38	
	>55	20 (4.7)	10.55	2.13	

Gender	Male	168 (39.3)	9.83	2.43	0.03
	Female	260 (60.7)	10.98	2.30	

Diabetes status	No	372 (8.9)	10.93	2.40	0.75
	Yes	56 (13.1)	10.36	2.40	

Education Level	Primary	25 (5.8)	10.15	2.60	0.32
	High school	140 (32.7)	10.80	2.47	
	University	214 (50.0)	11.01	2.27	
	Other	49 (11.4)	10.82	2.44	

Employment Status	Employed	179 (41.8)	10.98	2.52	0.61
	Not Employed or retired	249 (58.2)	10.76	2.20	

Insurance Status	Have insurance	106 (24.8)	11.08	2.59	0.23
	No Insurance	322 (75.2)	10.78	2.62	

Marital Status	Single	248 (57.9)	10.86	2.46	0.46
	Married	169 (39.5)	10.90	2.31	
	Divorced/Widow	11 (2.6)	11.10	1.74	

Nationality	Local	45 (10.5)	11.41	2.86	
	Arabs	206 (48.1)	11.01	2.27	0.04
	European	17 (4.0)	10.23	1.66	
	USA	18 (4.2)	10.80	1.76	
	South Asians	81 (18.9)	10.70	2.38	
	Others	61 (14.3)	10.30	2.63	

Smoking	Yes	88 (20.5))	10.43	2.51	
	No	340 (79.4)	10.97	2.41	0.01

Physically active	Yes	302 (70.6)	10.89	2.40	
	No	126 (29.4)	10.79	2.54	0.71

^*∗*^Using t-test or ANOVA analysis and significant at p≤0.05.

**Table 3:** Mean oral health behavior scores for participants.

Behavioral related questions	Observations	Mean	SD
How often do you smoke?	630	3.49	1.01
How often do you brush your teeth?	630	1.93	0.66
How often do you floss your teeth?	630	0.89	1.13
Have you received oral health information/instructions?	622	0.75	0.43
Did you follow the oral health instructions?	594	0.69	0.46
How often do you visit your dentist?	629	0.71	0.59
**Oral Health behavioral score**	**592**	**8.91**	**2.29**

**Table 4:** Relationship between demographic variables and mean oral health behavior score.

Variable	Categories	N (%)	Mean	SD	P value^*∗*^
Age	18-24	279(47.1)	8.38	2.27	
	25-34	132(22.3)	8.52	2.22	
	35-44	86(14.5)	9.14	2.36	0.04
	45-54	59(10.0)	8.56	2.19	
	>55	36(6.1)	8.24	2.49	

Gender	Male	251 (42.4)	7.86	2.50	≤0.001
	Female	341 (57.6)	9.24	2.06	

Diabetes Status	No	518 (87.5)	8.57	2.19	0.93
	Yes	74 (12.5)	8.25	2.52	

Education Level	Primary	30(5.1)	7.98	2.21	≤0.001
	High school	169(28.5)	8.17	2.33	
	University	330(55.7)	9.50	2.30	
	Other	63(10.6)	8.60	1.80	

Employment Status	Employed	292 (49.3)	8.86	2.29	0.04
	Not Employed or retired	300 (50.7)	8.10	2.17	

Insurance status	Have insurance	147 (24.8)	8.98	2.35	0.03
	No Insurance	445 (75.2)	8.15	2.25	

Marital Status	Single	298 (50.3)	8.37	2.27	0.03
	Married	276 (41.6)	8.76	2.28	
	Divorced/Widow	18 (3.0)	7.00	2.28	

Nationality	Local	62 (10.5)	9.20	2.86	0.02
	Arabs	296 (50.0)	8.30	2.27	
	European	30 (5.1)	8.85	1.66	
	USA	22 (3.7)	8.92	1.76	
	South Asian	115 (19.4)	8.50	2.38	
	Others	67 (11.3)	8.72	2.63	

Smoking	Yes	135 (22.8)	6.53	2.25	≤0.001
	No	457 (77.2)	9.12	1.86	

Physically Active	Yes	408 (68.9)	8.76	2.23	≤0.001
	No	184 (31.1)	8.02	2.17	

^*∗*^Using t-test or ANOVA analysis and significant at p≤0.05.

## Data Availability

The data used to support the findings of this study are included within the article.
