# Corrigendum to “Oral Health Knowledge and Behavior among Adults in the United Arab Emirates”

**DOI:** 10.1155/2019/3597303

**Published:** 2019-04-21

**Authors:** Eman Abu-Gharbieh, Basema Saddik, Mohammed El-Faramawi, Samer Hamidi, Mohammed Basheti

**Affiliations:** ^1^Clinical Sciences Department, College of Medicine, University of Sharjah, UAE; ^2^Department of Pharmacology and Toxicology, Dubai Pharmacy College, UAE; ^3^Department of Family and Community Medicine, College of Medicine, University of Sharjah, UAE; ^4^Department of Epidemiology, College of Public Health, University of Arkansas for Medical Sciences, USA; ^5^Department of Public Health, National Liver Institute, Menofiya University, Cairo, Egypt; ^6^School of Health and Environmental Studies, Hamdan Bin Mohammad Smart University, UAE; ^7^Faculty of Dentistry, Jordan University of Science and Technology, Jordan

In the article titled “Oral Health Knowledge and Behavior among Adults in the United Arab Emirates” [1], the name of the last author was given incorrectly as Mohammad Basheti. The author's name should have been written as Mohammed Basheti. The revised authors' list is shown above.
